# Transcriptomic analysis of the black tiger shrimp (*Penaeus monodon*) reveals insights into immune development in their early life stages

**DOI:** 10.1038/s41598-021-93364-9

**Published:** 2021-07-06

**Authors:** Pacharaporn Angthong, Tanaporn Uengwetwanit, Sopacha Arayamethakorn, Wanilada Rungrassamee

**Affiliations:** National Center for Genetic Engineering and Biotechnology, National Science and Development Agency, 113 Thailand Science Park, Phahonyothin Road, Khlong Luang, Pathum Thani 12120 Thailand

**Keywords:** Molecular biology, Transcriptomics

## Abstract

With the rapid growth in the global demand, the shrimp industry needs integrated approaches for sustainable production. A high-quality shrimp larva is one of the crucial key requirements to maximize shrimp production. Survival and growth rates during larval development are often criteria to evaluate larval quality, however many aspects of gene regulation during shrimp larval development have not yet been identified. To further our understanding of biological processes in their early life, transcriptomic analysis of larval developmental stages (nauplius, zoea, mysis, and postlarva) were determined in the black tiger shrimp, *Penaeus monodon* using next-generation RNA sequencing. Gene clustering and gene enrichment analyses revealed that most of the transcripts were mainly related to metabolic processes, cell and growth development, and immune system. Interestingly, Spätzle and Toll receptors were found in nauplius stage, providing evidence that Toll pathway was a baseline immune system established in early larval stages. Genes encoding pathogen pattern-recognition proteins (*LGBP*, *PL5-2* and *c-type lectin*), prophenoloxidase system (*PPAE2*, *PPAF2 *and *serpin*), antimicrobial peptides (*crustin *and *antiviral protein*), blood clotting system (*hemolymph clottable protein*) and heat shock protein (*HSP70*) were expressed as they developed further, suggesting that these immune defense mechanisms were established in later larval stages.

## Introduction

The black tiger shrimp (*Penaeus monodon*) is one of the economically important shrimp species with high global market demand^1^. However, sustainable production of the black tiger shrimp is still difficult due to various factors such as lack of selective breeding programs and effective disease control approaches^2,3^. The major shrimp diseases are mostly caused by bacterial, viral and fungal pathogens^4,5^ such as white spot syndrome virus (WSSV)^4,6^, the microsporidian *Enterocytozoon hepatopenaei* (EHP)^7^, and *Vibrio parahaemolyticus* (acute hepatopancreatic necrosis (AHPND) pathogen)^8,9^, resulting in mass mortalities in shrimp. Importantly, early developmental stages of animals have been reported for a higher infection risk by pathogenic microorganisms than adult stage^10,11^. In shrimp, AHPND causes severe mortality in early stages of shrimp including *P. monodon*^12–14^. Additionally, the infectious hypodermal and hematopoietic necrosis virus (IHHNV) causing runt deformity syndrome (RDS) and EHP can infect shrimp at the early life stage which can further transmit to later shrimp developmental stages, resulting in poor survival rates or growth performance in shrimp aquaculture^15,16^. Consequently, a selection of high-quality shrimp larva based on their survival, stress resistance and growth performance has become one of the important aspects to lower risks of disease outbreak in grow-out pond systems. An understanding of biological processes including growth, immune and stress-related pathways underlining early larval stages in shrimp will provide an essential foundation for identifying developmentally important genes useful for future larval quality screening.

Being a crustacean, shrimp possesses an exoskeleton surface and undergoes metamorphosis through the following stages: egg, nauplius, zoea, mysis, postlarva, juvenile and adult^17,18^. Their morphology, physiology and ecology are drastically transformed during the early life stages. To understand biological and physiological processes at larval stages, differential gene expression analysis was carried out in various crustaceans such as Pacific white shrimp (*P. vannamei*)^19,20^, water flea (*Daphnia magna*)^21^, barnacle (*Amphibalanus amphitrite*)^22^ and crab (*Portunus pelagicus*)^23^. Molting- and exoskeleton developmentally-related genes are mostly found associated in early developmental stages of Pacific white shrimp^24^ and crab^23^ during their metamorphosis. However, the functional genes involved in physiological changes during the early life stages of *P. monodon* have not yet been characterized. In addition to growth- and metabolic-related processes, the immune system in the early life of shrimp undergoes rapid changes and becomes more established as they develop further^25^. Shrimp rely on their innate immune system for defense and protection against pathogens^26,27^. The innate immune system consisting of cellular and humoral immune responses that interact to recognize and eliminate invading microorganisms^28^. The cellular immune responses mostly occur in hemocytes, which recognize the component on a cell of microorganism via pattern recognition proteins (PRPs) and trigger a series of immune responses such as phagocytosis, nodulation and encapsulation^27,29,30^. In contrast, the humoral immune responses are found in hemolymph such as prophenoloxidase (proPO) system, blood clotting system and antimicrobial peptides (AMPs)^27,31,32^. Several studies have been conducted on shrimp immune responses in different experimental conditions. For instance, investigation on the transcriptional profile of immune-related genes under AHPND, *V. harveyi* or WSSV infection^33–36^. However, most of these studies were identified in juvenile and adult shrimp stages, and there is still limited understanding on the development of innate immune system in early life stages.

Here, we aimed to understand gene expression profiles, particularly those related to immune and stress responses in the early developmental stages of *P. monodon*. The four larval stages in shrimp (nauplius, zoea, mysis and 15-day-old postlarva) were collected for transcriptomic analysis. Our findings on the biological processes and immune responses will contribute to understanding molecular mechanisms in shrimp at their early life stages, and further studies in areas of functional gene analysis and developmental biology. Ultimately, this work can be implemented with a strategic approach to design efficient shrimp feeds and practices to reduce the risk of diseases and increase farm productivity.

## Results

### De novo assembly and functional annotation

**Figure 1:**
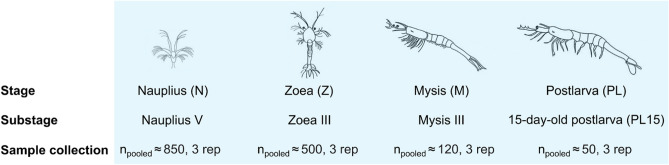
Schematic diagram of larval stages during early development of *P. monodon* collection. Shrimp samples were collected at stages of nauplius (N), zoea (Z), mysis (M) and 15-day-old postlarva (PL15) for transcriptome analysis.

**Figure 2:**
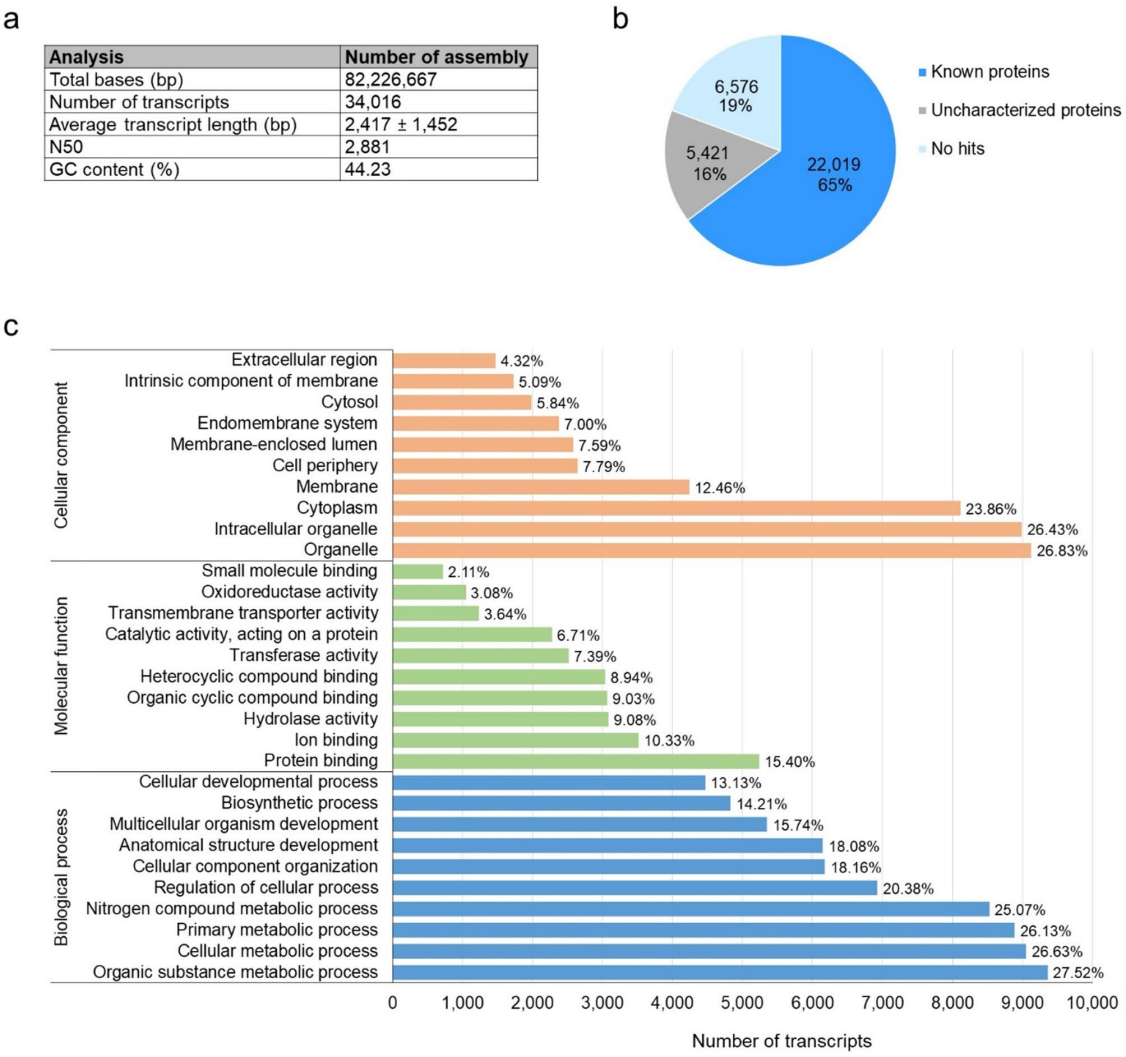
Summary of assembly and annotation of *P. monodon* at early developmental stages, including nauplius, zoea, mysis and 15-day-old postlarva. (**a**) Summary of read depth of RNAseq data. (**b**) The pie chart shows percentage of transcripts matched sequences in NCBI RefSeq database. (**c**) Histogram gene ontology classification of the transcripts into three main categories including biological process, molecular function and cellular component.

To determine gene expression profiles in *P. monodon* at early life stages, cDNA libraries were constructed for transcriptomic analysis in the four developmental stages (nauplius (N), zoea (Z), mysis (M) and 15-day-old postlarva (PL15)) (Fig. 1). After quality assessment and data filtering, an average of 403,006,878 ± 4,628,572 clean paired-end reads were used for further analysis. A total of 34,016 transcripts were assembled with a total length of 82,226,667 bp, an average length of 2,417 ± 1,452 bases, a 50% total assembly length (N50) of 2,881 bp, and a GC content of 44.23% (Fig. 2a). Transcriptome assembly validation was done using Benchmarking Universal Single-Copy Ortholog (BUSCO)^37^, which showed 90.98% of complete BUSCO indicating the high quality of assembly. All transcripts obtained were annotated by comparison with the reference protein database (RefSeq) and GO database, in which 22,019 (65%) transcripts were annotated (Fig. 2b), no hits and unknown functions were 19% and 16%, respectively. GO annotations were assigned to three principal GO databases classification. The main ontologies represented were (1) 43.34% biological processes, (2) 37.11% molecular functions and (3) 39.45% cellular functions (Fig. 2c). The top GO term under biological processes was from organic substance metabolic processes (27.52%), cellular metabolic processes (26.63%) and primary metabolic processes (26.13%). The top GO term under molecular function was protein binding (15.40%), ion binding (10.33%) and hydrolase activity (9.08%). The most prevalent GO term in cellular component was organelle (26.83%), intracellular organelle (26.43%) and cytoplasm (23.86%). The GO term distribution indicating that our RNA sequencing analysis yielded a good coverage of gene expression in shrimp at early life stages.

### Clustering of gene expression profiles across shrimp growth stages and enrichment analysis

To determine the gene expression dynamics in the early life of *P. monodon*, gene expression clustering was performed to categorize expression patterns associated with the developmental stages (Fig. S1). Our gene enrichment analysis revealed transcripts with their function in various biological processes (i.e., signal transduction, cell cycle, RNA polymerase II transcription, post-translational protein modification), metabolic pathways (i.e., metabolisms, metabolism of proteins and metabolism of carbohydrates) and developmental biological pathways and immune system in all stages. Gene expression patterns were grouped into 9 clusters. Cluster 1 to 4 showed highly expressed genes in each life stage, namely nauplius (1062 transcripts), zoea (71 transcripts), mysis (139 transcripts) and PL15 (1,146 transcripts). Cluster 5 (2554 transcripts) and 6 (423 transcripts) showed decreasing gene expression patterns with developmental stages, while cluster 7 (3780 transcripts) and 8 (1237 transcripts) showed increasing expression patterns from the nauplius stage to the later growth stage. Cluster 9 (9870 transcripts) was a group of transcripts with unchanged expression levels throughout early developmental stages.

### Expression profiles of genes related to metabolic pathways and growth development in early life stages of *P. monodon*

**Figure 3:**
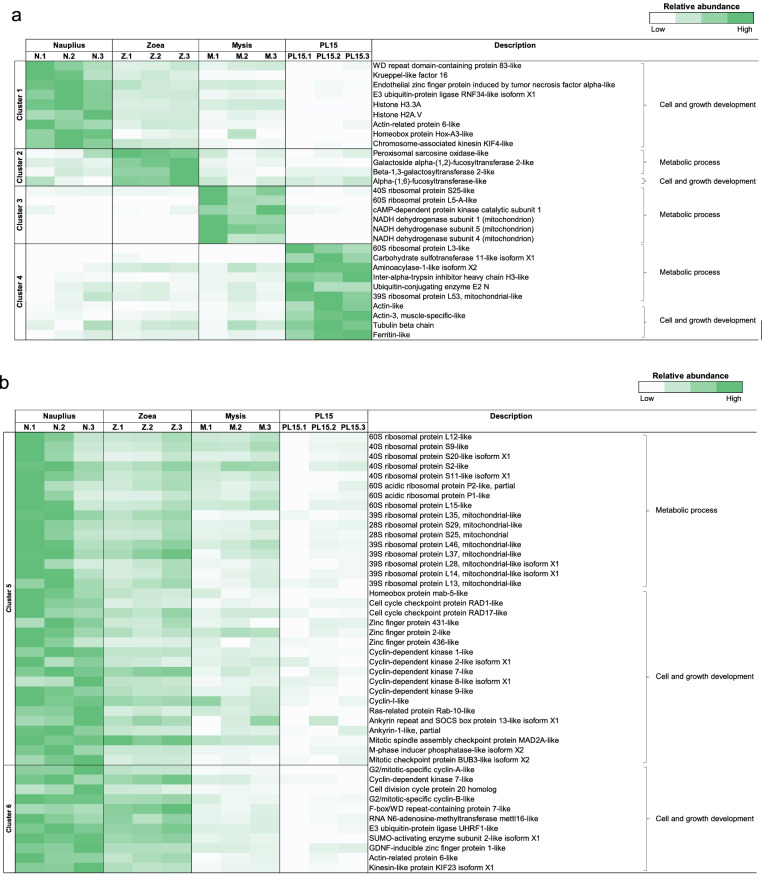

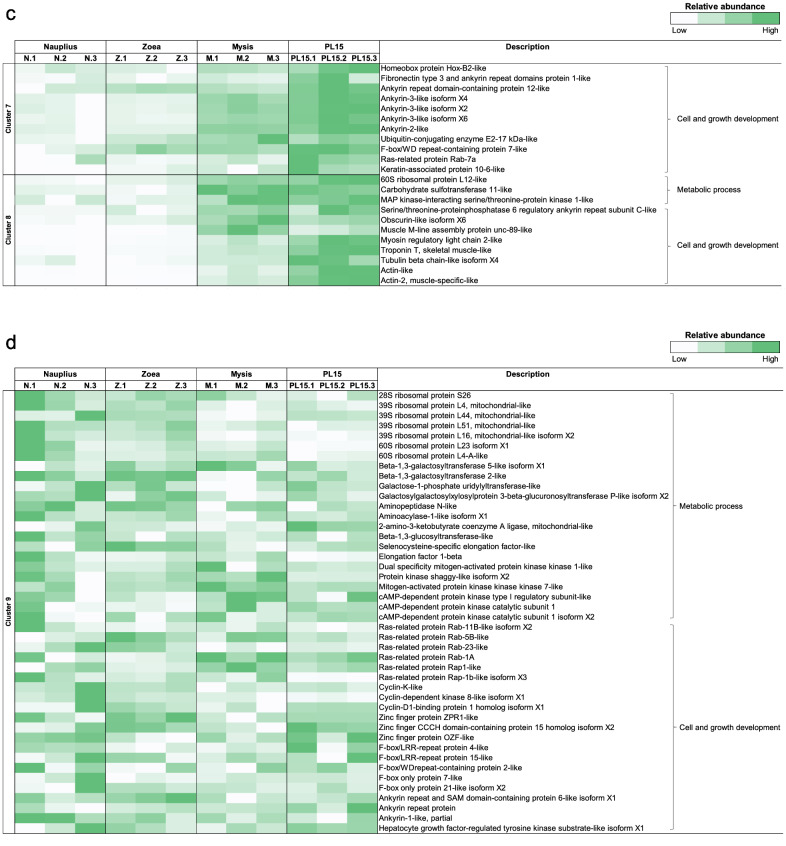
Heatmap showing the enriched transcripts involved in metabolic processes and cell and growth development associated with early shrimp stages, which were divided to nauplius (N), zoea (Z), mysis (M) and 15-day-old postlarva (PL15). (**a**) Cluster 1–4 shows the pattern of highly gene expressed profiles in nauplius, zoea, mysis and 15-day-old postlarva. (**b**) Cluster 5–6 shows decreasing pattern of gene expression from nauplius to postlarval stage, (**c**) cluster 7–8 shows increasing pattern of gene expression from nauplius to postlarval stage, and (**d**) cluster 9 shows the pattern of unchanged in gene expression across all life stages.

Here, expression levels of transcript-related to metabolic processes, and cell and growth development were identified in larval stages (Fig. 3 and Table S1). A group of transcripts was highly expressed and specific to each larval shrimp stage (Fig. 3a). For instance, the transcripts involved in cell and growth development such as *Krüppel-like factor*, *zinc finger protein*, *histone* and *homeobox protein* were enriched in the nauplius stage (Cluster 1), while *alpha (1,6)-fucosyltransferase* was highly expressed in the zoea stage (Cluster 2). Moreover, the PL15 stage (Cluster 4) showed increased expression levels of *actin*, *ferritin* and *tubulin* transcripts. On the other hand, transcripts-related with metabolic pathways such as *galactoside alpha-(1,2)-fucosyltransferase* in zoea, *cAMP-dependent protein kinase catalytic* and *NADH dehydrogenase* in mysis (Cluster 3), and *carbohydrate sulfotransferase* in PL15 stage. These results show that the dynamic changes of distinct classes of genes during the development reflecting different needs of gene functions for their metabolic processes and maintenance of their cellular functions.

To further explore changes in metabolisms and growth-related pathways under shrimp metamorphosis, the transcripts with continuously decreasing expression patterns (Cluster 5) were mostly members of a ribosomal protein family (Fig. 3b). The higher numbers of various components of ribosomal protein transcripts were found in nauplii, suggesting different physiological and metabolic activities in each shrimp’s life stage. Moreover, transcripts encoding *cyclins* and *zinc finger proteins* which play important roles in cell cycle and growth development were decreasing as shrimp developed from nauplius to PL15 stage (Cluster 5). The transcript involved in cell and growth development such as *cell division cycle protein* and *kinesin-like protein* showed a decreasing trend once shrimp developed into the zoea stage (Cluster 6) (Fig. 3b). On the other hand, cell growth and development-related transcripts such as *ankyrin*, *muscle M-line assembly protein*, *obscurin*, *tubulin*, *keratin* and *actin* were increasing (Cluster 7 and 8) (Fig. 3c), indicating that they were essential in shrimp as they further developed.

In addition, there were a group of unchanged transcripts levels across the four developmental stages (cluster 9) (Fig. 3d). These were those related to metabolic processes (*ribosomal protein* family, *elongation factors*, *mitogen-activated protein kinase*) and cell and growth development (*ras-related proteins*, *cyclins*, *zinc finger proteins*, *F-box proteins* and *ankyrin*) were constitutively expressed during the growth development, indicating that these genes were required for maintenance of basic cellular functions and developmental processes in shrimp.

### Expression profiles of genes related to immune responsive genes in early life stages of *P. monodon*

**Figure 4:**
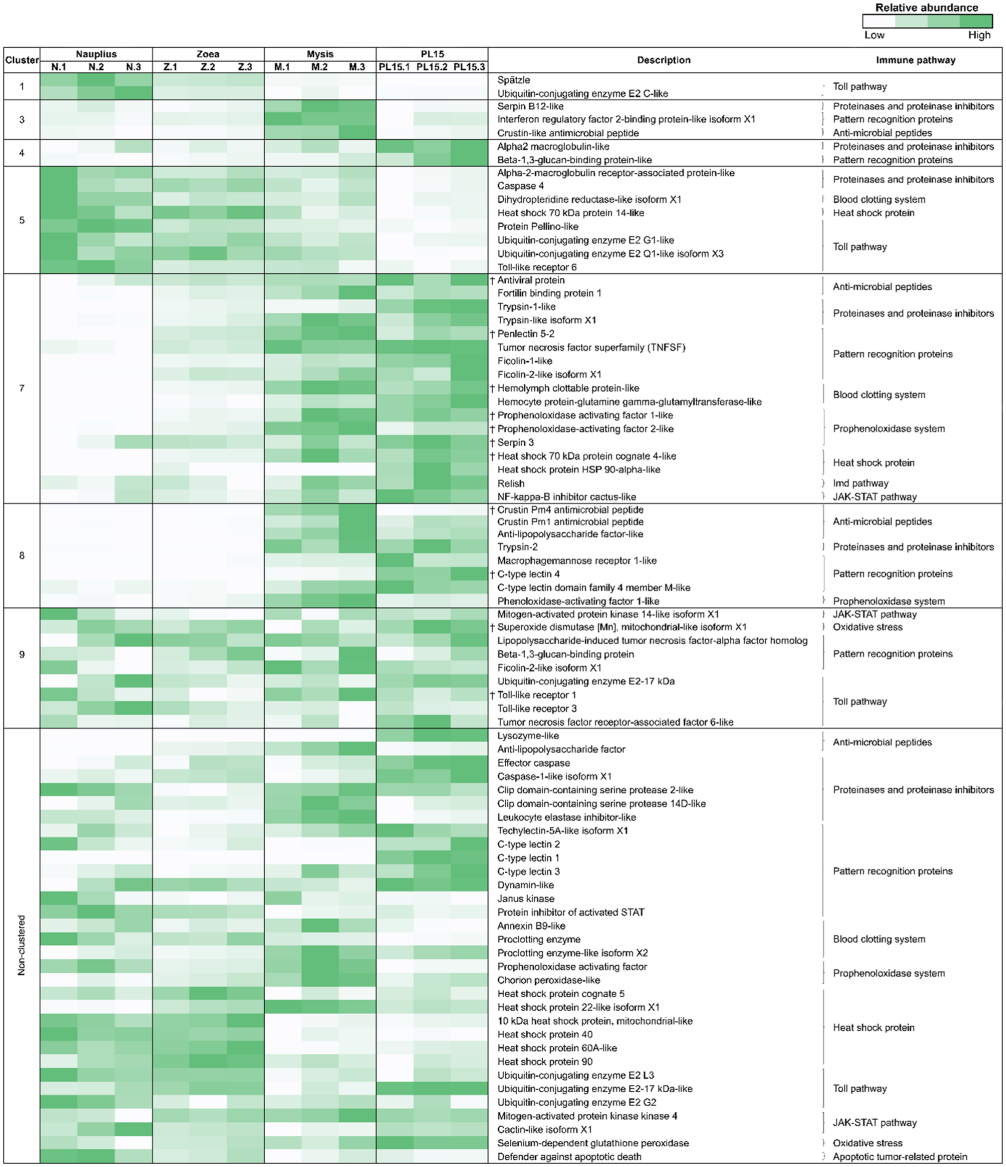
Heatmap showing the enriched transcripts involved in immune responses in early life stages of black tiger shrimp, including nauplius (N), zoea (Z), mysis (M) and 15-day-old postlarva (PL15). The dagger (†) indicates genes that were further validated by quantitative real-time PCR.

To understand immune development in shrimp, the expression dynamics of immune-related transcripts during the developmental stages of shrimp were explored (Fig. 4). Here, we identified immune-related genes including Toll pathway, immune deficiency (IMD) pathway prophenoloxidase system (proPO system), pattern recognition proteins (PRPs), blood clotting system, antimicrobial peptides (AMPs), heat shock proteins (HSPs), proteinases and proteinase inhibitors and oxidative stress in shrimp immune network in each stage. For instance, the immune-related genes involved in the Toll pathway (*Spätzle* and *ubiquitin-conjugating enzyme*) were significantly expressed in the nauplius stage (cluster 1), suggesting a baseline defense mechanism to protect themselves when they first hatched out from eggs. Moreover, the *crustin-like antimicrobial peptide* was found in higher abundance in mysis stage (cluster 3), while *alpha 2 macroglobulin* and *beta-1,3-glucan binding protein* (*BGBP*) showed higher expression levels in PL15 shrimp (cluster 4). Components of Toll pathway (*protein pellino*, *Toll-like receptor 6* (*TLR6*) and *ubiquitin-conjugating enzymes*), proteinases and proteinase inhibitors (*caspase4*), blood clotting system (*dihydropteridine reductase isoform X1*) and heat shock protein showed higher transcript levels in the nauplius stage and decreasing in later stages (cluster 5). Conversely, AMPs (*antiviral protein*, *fortilin binding protein, crustin Pm1*, *crustin Pm4* and *anti-lipopolysaccharide factor* (*ALF*)), PRPs (*penlectin5-2* (*PL5-2*), *tumor necrosis factor ligand*, *ficolins*, *macrophage mannose receptor* and *c-type lectins*), IMD pathway (*Relish*), proPO system (*prophenoloxidase-activating factor 1* (*PPAF1*), *PPAF2* and *serpin3*), blood clotting system (*hemolymph clottable protein*), heat shock proteins (*HSP70* and *HSP90*) and JAK-STAT pathway (*NF-kappa-B inhibitor cactus*) were expressed in an increasing manner from nauplius to postlarval stage (cluster 7 and 8). The immune-related transcripts with unchanged expression levels (cluster 9) throughout the four early life stages were those related to oxidative stress response (*superoxide dismutase (Mn), mitochondrial-like isoform X1* (*MnSOD*)), PRPs (*BGBP*, *lipopolysaccharide-induced tumor necrosis factor-alpha factor homolog* (*LITAF*) and *ficolin-2-like isoform X1*) and Toll pathway (*Toll-like receptor 1* (*TLR1)*, *Toll-like receptor 3* (*TLR3* and *tumor necrosis factor receptor-associated factor 6* (*TRAF6*)), suggesting for their important roles in maintaining homeostasis during their growth development. Moreover, there were additional 32 non-clustered transcripts with their roles related to AMPs, proteinases and proteinase inhibitors, PRPs, blood clotting system, proPO system, HSPs, Toll pathway, JAK-STAT pathway, oxidative stress and apoptotic tumor-related protein were found as well. Our results showed evidence of shrimp immune-related genes expressed in early life stages, in which more components of the shrimp immune system were expressed in the later stages under non-pathogenic rearing conditions.

**Figure 5:**
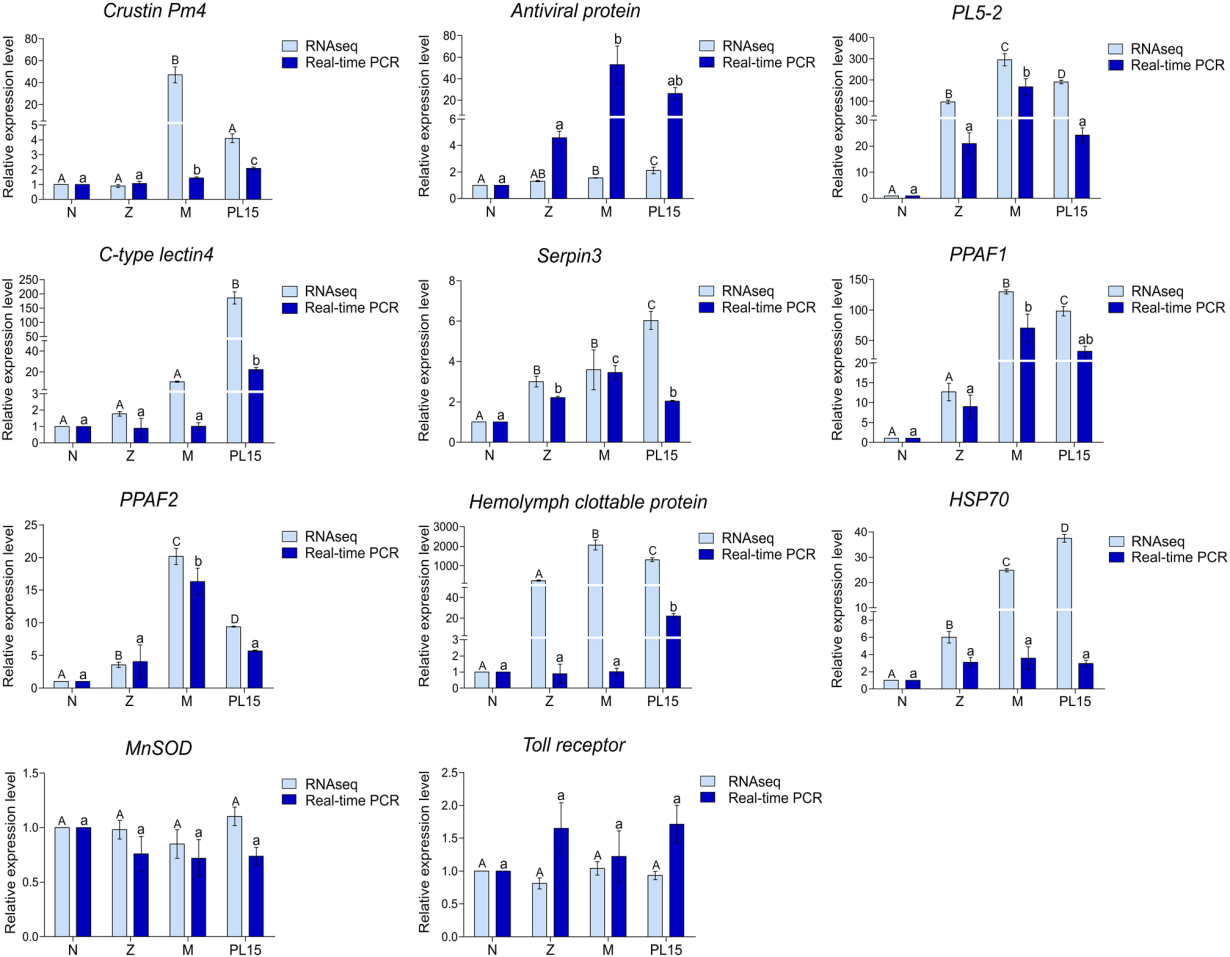
Validation of RNAseq data by quantitative real-time PCR (qPCR) of immune-related transcripts including *crustin Pm4*, *antiviral protein*, *penlectin 5–2* (*PL5-2*), *c-type lectin 4*, *serpin 3*, *prophenoloxidase-activating factor 1* (*PPAF1*), *prophenoloxidase-activating factor 2* (*PPAF2*), *hemolymph clottable protein*, *heat shock protein 70* (*HSP70*), *superoxide dismutase* (*Mn*) (*MnSOD*) and *toll-like receptor* in nauplius (N), zoea (Z), mysis (M) and 15-day-old postlarva (PL15). The error bars indicate standard error of the mean from biological triplicates. Different letters show significant different by ANOVA (*p value* < 0.05).

To validate our transcriptomic profiles, 11 immune-related genes (*crustin Pm4*, *antiviral protein*, *PL5-2*, *c-type lectin4*, *serpin3*, *PPAF1*, *PPAF2*, *hemolymph clottable protein*, *HSP70*, *MnSOD* and *TLR1*) were selected for gene expression analysis using real-time PCR (Fig. 5). The expression patterns of our target immune-related genes were consistent to gene expression profiles obtained from the next-generation sequencing (Fig. 4), except *HSP70*. Although the expression level of *HSP70* was not statistically significantly different among shrimp growth stages, *HSP70* transcript was expressed in higher abundance in zoea, mysis and PL15 than the nauplius stage.

### Evidence of immune development in their early life stages

**Figure 6:**
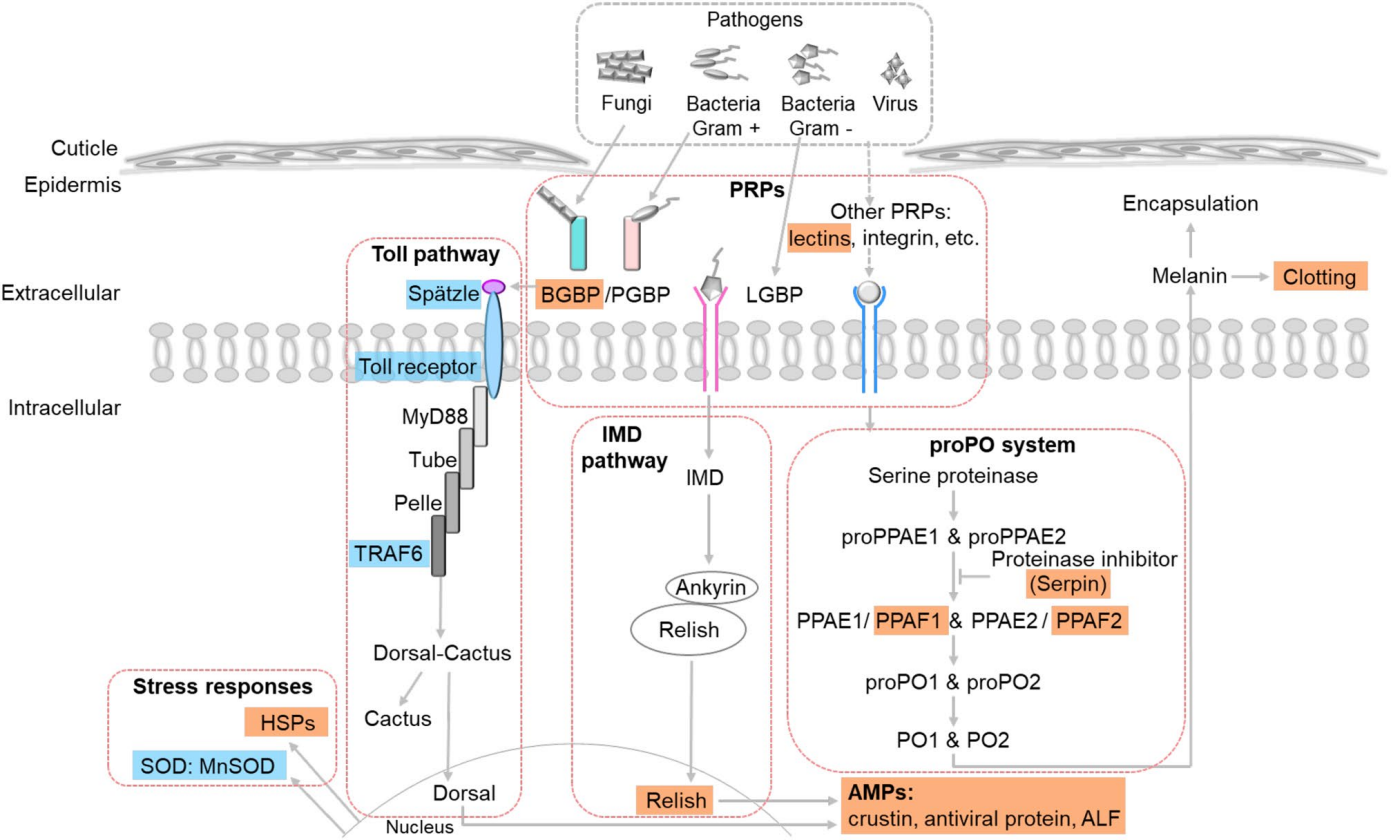
Schematic model of shrimp immune system in early life stages of *P. monodon*, including nauplius, zoea, mysis and 15-day-old postlarva. Pattern recognition proteins (PRPs) (beta 1,3-glucan binding protein (BGBP), lipopolysaccharide and beta 1,3-glucan binding protein (LGBP) and lectin), prophenoloxidase system (proPO system) (prophenoloxidase-activating factor 1 (PPAF1), prophenoloxidase-activating factor 2 (PPAF2) and serpin), antimicrobial peptides (AMPs) (crustin, anti-lipopolysaccharide factor (ALF) and antiviral protein), blood clotting system, Toll pathway (toll receptor, Spätzle and tumor necrosis factor receptor-associated factor 6 (TRAF6)), IMD pathway (Relish) and stress responses (heat shock proteins (HSPs) and manganese superoxide dismutase (MnSOD) were involved in early life stages of shrimp. Blue box represents transcript expression found associated with the four larval stages, while orange box represents an immune gene with increasing expression pattern during the development. The compotents in shrimp immune pathways were adapted from previous studies^31^.

After hatching out from eggs, shrimp go through series of changes such as nutrient requirement, energy consumption and feeding behavior, therefore shrimp become more exposed to rearing environments as they develop. Shrimp directly interact and exchange with a variety of microorganisms in their rearing environment, and they rely mainly on innate immune responses as their defense mechanisms during the development. Here, our transcriptomic analysis revealed components of innate immune-related pathways associated with each life stage (Fig. 6). The schematic model showed constitutive expression of immune-related genes identified in larval stages such as *Spätzle*, *TLR1*, *TLR6*, *TRAF6* and *MnSOD*. Toll-Spätzle complex acts as pathogen recognition and triggers Toll signaling pathway for series of immune responses to eliminate the pathogens^38^. Our findings suggest that Toll pathway was a baseline immune system established in early larval stages. On the other hand, genes encoding PRPs (*PL5-2*, *c-type lectins* and *ficolins*), proPO system (*PPAF1*, *PPAF2* and *serpin3*), IMD pathway (*Relish*) AMPs (*crustin Pm1, crustin Pm4*, *ALF* and *antiviral protein*), blood clotting system (*hemolymph clottable protein*) and HSPs (*HSP70* and *HSP90*) were expressed in later growth stages.

## Discussion

Transcriptomic analysis has been widely applied in aquaculture to understand underlying molecular mechanisms related to various biological processes^24,39,40^. In penaeid shrimp, gene expression profiles related to growth, metabolic activities and immune responses have mostly been identified in juvenile and adult stages^33,35,41^, while little is still known in shrimp at early life stages including nauplius, zoea, mysis and postlarva. Particularly, their morphological and physiological were dramatically changed from nauplii into postlarva in their early life, and they can be more susceptible to diseases due to their underdeveloped immune systems^17^. Here, we determined gene expression profiles of *P. monodon* in the early life stages to further our understanding of the biological processes and mechanisms related to their early development, providing insights into shrimp immune development and growth-related pathways.

In this study, the enriched pathways indicating life-stage specific expression patterns in the early developmental stages of *P. monodon* were mainly related to metabolic processes, immune system, and cell and growth development. In particular, several transcript-related to ontogenetic developmental processes were significantly expressed in each life stage such as *homeobox proteins*, *cyclin-dependent kinase*, *zinc finger proteins*, and *histone-related proteins*. Among those, homeobox proteins are essential for regulating the development of body plan of various species including humans^42^, flies^43,44^ and worms^45^. Thus, the higher transcript levels of homeobox proteins in early life stages suggest their relevant roles in morphological transformation of body structures such as appendage, organ and segment of shrimp. Additionally, cyclins, which play an important role in cell cycling by interacting with *cyclin-dependent kinases* showed a decreasing trend as shrimp developed from nauplii to PL15. On the other hand, we found a group of transcripts related to the molting process and exoskeleton formation was increasing with developmental stages including *ferritin*, *tubulin* and *keratin*^23,46,47^. This was congruent with the previous report in Pacific white shrimp (*P. vannamei*)^46^. Being a crustacean with their body surface covered by exoskeleton shell^48,49^, shrimp periodically undergo a molting process to grow and this process requires a newly synthesized exoskeleton layer^50,51^, hence, the transcript-related to this process were crucial and associated to shrimp developmental process. Moreover, *ankyrin*, *obscurin* and *actin*, which play essential roles in muscle development were highly expressed in PL15. In this process, ankyrin will bind to obscurin in the sarcoplasmic reticulum and lead to muscle growth development^52,53^. Obscurin-knockout mice show a decreasing level of ankyrin expression, contributing to the loss of muscle mass^54^. Actins play roles in microfilament formation in muscles and serve as the major component of muscle during the molting cycle in crustaceans^24,55^. Our results showed that these genes were important for growth development in shrimp.

Ribosomal proteins are composed of two asymmetric subunits forming into a complex and play an important part in protein translation, which are essential for cell growth and development^56,57^. Ribosomal proteins can be remarkably different between organisms, developmental stages and growth conditions^57^. Here, we identified various forms of the ribosomal proteins associated with shrimp developmental stages, in which some forms were found in higher abundance in nauplii, suggesting for the specific function required for early life. Previous studies have reported on increased expression levels of various ribosomal proteins during the embryonic development of zebrafish (*Danio rerio*)^58,59^ and bighead carp (*Hypophthalmichthys nobilis*)^60^, and this could be explained by higher rates of cell division and cell differentiation taking place during the growth development. On the other hand, *elongation factors* and *mitogen-activated protein kinase*, known housekeeping genes in shrimp showed constitutively expressed during early life stages. Housekeeping genes are required for the basic functions of the cell, and they are expressed in all cells of an organism^61^. Thus, shrimp larva needed a group of these genes for maintenance of their cellular function across developmental stages.

In addition to growth and developmental processes, there were some immune-related transcripts associated with the shrimp developmental stage. Several major shrimp immune responses such as pattern-recognition proteins (PRPs), prophenoloxidase system (proPO system), immune deficiency (IMD) pathway and antimicrobial peptides (AMPs) showed increasing expression patterns during developmental stages. In crustaceans, PRPs play important role in detecting pathogen-associated molecular patterns (PAMPs) presented on the surface of microorganisms and activate downstream immune responses such as AMPs^27,62^, proPO system^63,64^, melanization and blood coagulation in arthropods^65,66^. The proPO system is one of the main shrimp innate immune response and is initiated by the binding of PRPs to microbial membrane components such as peptidoglycans (PGs) and lipopolysaccharide (LPS)^63^. The complex leads to the activation of serine proteinase cascades to cleave proPO, generating active phenoloxidase (PO) to activate melanization process^63,64,67^. Here, transcripts of *PPAF1*, *PPAF2* and *serpin3,* members of proPO system were increased with larval developmental stages. Consistently, the expression level of proPO has been reported to gradually increase during the development of bivalve molluscs (*Crassostrea gigas*, *Argopecten ventricosus* and *Nodipecten subnodosus*)^68^ and black tiger shrimp (*P. monodon*)^25^. In *P. monodon*, the transcript level of proPO was lower in nauplii and was gradually increased as shrimp developed further into zoea and postlarval stages^25^. Similarly, the expression level of *Relish* was also increasing with the larval developmental stage. Relish is a key transcription factor in IMD pathway that regulates the expression of AMPs in *Drosophila*^69^ and crustaceans such as shrimp^31,70^. In penaeid shrimp, AMPs are part of important host immune systems, in which they play antimicrobial activity against Gram-positive and Gram-negative bacteria, fungi, yeasts and viruses^31,71^. The transcript levels of *crustin Pm1, crustin Pm4*, *anti-lipopolysaccharide factor* (ALF) and *antiviral protein,* members of AMP families, were increased with shrimp developmental stage, and our observations were consistent with the previous studies in *P. monodon* and *P. vannamei* larval and juveniles^25,71,72^. In *P. monodon*, crustin Pm1 has been shown to inhibit only the growth of Gram-positive bacteria (*Staphylococcus aureus* and *Streptococcus iniae*)^73^, while crustin Pm4 exhibits antimicrobial activity against both Gram-positive bacteria (*Bacillus megaterium*) and Gram-negative bacteria (*Escherichia coli* and *V. harveyi*)^74^. Additionally, the transcript level of *crustin Pm4* in *P. monodon* is inducible after WSSV infection suggesting that crustin Pm4 might play a protective role against viral pathogen^74^. Moreover, the previous study has reported that the antiviral protein of *P. monodon* showed strong antiviral activity against WSSV^75^. Among AMP families, ALF has been reported to exhibit broad-spectrum activity against various microorganisms including bacteria, fungi and viruses^76^. In ALF-knockout *P. vannamei*, the exposure to pathogenic bacteria (*V. penaeicida*) or fungi, (*Fusarium oxysporum*) resulted in high mortality, providing evidence that ALF can protect shrimp against different microbial infection^77^. It is worth noting that diverse types of AMPs were expressed in association with early larval stages. Having different AMPs with broad-spectrum antimicrobial activities as key effectors might help to provide immune protection during larval development.

Toll pathway plays a crucial role in response to Gram-positive bacteria, fungi and viruses. Toll receptors recognize a presence of a pathogen, leading to activation of signaling proteins including Myeloid differentiation factor 88 (MyD88), Tube, Pelle and tumor necrosis factor receptor-associated factor 6 (TRAF6), relaying the signal to the Dorsal-Cactus complex. Cactus is phosphorylated, dislocated from Dorsal, and degraded, while NF-κB transcription factor Dorsal translocated into the nucleus to activate the expression of AMPs^28,78^. In *P. monodon*, Toll-like receptors (TLRs) have been reported as part of shrimp defense mechanisms against pathogen invasion^79,80^. Expression of *TLR* transcripts of *P. monodon* larva and adult are inducible upon exposure to *V. harveyi*^80,81^, suggesting their involvement in activating shrimp immune responses against pathogenic bacteria. Interestingly, TLRs have been reported to be constitutively expressed in shrimp tissues of *P. vannamei*^82^ and *Fenneropenaeus chinensis*^83^ under the non-pathogenic condition as well, indicating that they also serve as part of primary innate immune responses in shrimp. Here, we identified three isoforms of TLRs, *TLR1*, *TLR3* and *TLR6* in *P. monodon* larva. Among the three identified toll isoforms, transcript levels of *TLR6* showed a decreasing trend, while *TLR1* and *TLR3* were constitutively expressed with shrimp larval development. Different toll-like receptor isoforms such as *TLR1* and *TLR3* have been identified *P. vannamei*^82,84^, and they are inducible upon a pathogen exposure in *P. vannamei*^82,84^. However, the function and importance of different TLR isoforms of *P. monodon* have not been addressed and need to be further characterized. In addition to *TLRs*, *TRAF6*, one of the core components of Toll pathway also showed constitutive expression in early developmental stages. Previous studies have reported an increased expression level of *TRAF6* in *P. monodon* post-larvae and adult after *V. harveyi* exposure^80,81^. Our results show that several components of Toll signaling pathway were established since the nauplius stage, suggesting that Toll pathway was primary immune response system early in life of *P. monodon*. Toll signaling pathway could provide a baseline immune response for host defense mechanisms against invading pathogens in their early larval stages.

In conclusion, we provide the first report on gene expression dynamics in the early development of *P. monodon*. The pathway enrichment and gene clustering analyses showed expression patterns of the transcript related to various biological processes such as metabolism, cell and growth development and immune response systems, reflecting different activities taking place at each life stage. In particular, we provide evidence of innate immune presence in early larval development such as Toll signaling pathway, proPO system, AMPs, and PRPs. Understanding developmental dynamics at molecular levels including relevant biological processes and immune response system of *P. monodon* at early life will lead to the future development of efficient feeds and immunostimulatory additives suitable for each developmental stage. The fundamental knowledge of biological processes can be further applied for larval quality screening.

## Methods

### Shrimp samples collection

The black tiger shrimp (*P. monodon*) were reared at Shrimp Genetic Improvement Center (SGIC), National Science and Technology Development Agency (NSTDA) in Surat Thani province, Thailand. Shrimp were unfed at nauplius stage. At zoea stage, larvae were fed with microalgae *Thalassiosira* sp. and *Chaetoceros* sp. The heat-treated *Artemia* and microalgae were fed to mysis shrimp. After they reached postlarval stage, live *Artemia* and commercial feed diets were given to shrimp. Shrimp larval stages were identified based on morphological classification of *P. monodon* larva^85^ under stereo microscope. Each larval stage was collected at the same sampling period and in triplicates from three independent shrimp families at our breeding facility (Fig. 1). Briefly, nauplii (n_pooled_ ≈ 850) were collected when they reached substage V, while zoea (n_pooled_ ≈ 500) and mysis (n_pooled_ ≈ 120) were collected when they reached their substage III. Postlarva PL15 (n_pooled_ ≈ 50) were collected when they reached 15-day-old. Shrimp samples were immediately snap-frozen in liquid nitrogen and stored at − 80 °C until used.

### RNA extraction and library preparation

Total RNA from each shrimp sample was extracted by using TRI Reagent (Molecular Research Center, USA) according to manufacturer’s protocol. Each pooled shrimp sample was immediately grounded in a mortar containing liquid nitrogen. An equal amount of 50 mg from each ground tissue sample was homogenized in TRI Reagent and subjected to chloroform extraction. DNA contamination in RNA samples were removed by treating RNA with RQ1 RNase-free DNase (Promega, USA). RNA purity and concentration were analyzed by NanoDrop (ND-8000) spectrophotometer, and the quality of RNA was examined under 1% agarose gel electrophoresis. DNA-free RNA samples were subjected to Illumina sequencing service at Macrogen Inc. (Korea). RNA sequencing libraries were prepared using the TruSeq Stranded mRNA LT Sample Prep kit (Illumina, USA) following manufacturer’s protocol. Each library was subjected to 150 bp paired-end sequencing using NovaSeq 6000 (Illumina, USA).

### Transcriptome data analysis

Quality of the raw RNA-Seq data were processed through FASTQC^86^ and TrimGalore (https://github.com/FelixKrueger/TrimGalore) to remove adaptor-sequences and read ends of low base quality (Phread score < 20). The reads with at least 100 bp in length were used in downstream data analysis. Sequencing reads were assembled by using Trinity with default parameters^87^. The longest isoform for each gene was selected. The assembled contigs were merged with the published full-length transcript sequences^88^ according to a similarity criterion of 98% and 80% minimal alignment coverage for the shorter sequence using CD-HIT^89^. The non-redundant reference sequences were used in downstream differential gene expression analysis and functional annotation. The transcripts were evaluated by using BUSCO with default settings and BUSCO v3.0.2 core dataset for single-copy conserved eukaryotic genes^37^. Functional annotations were carried out by using BLASTX against the NCBI protein reference database (Refseq)^90^ which including the proteins from a reference *P. monodon* genome^91^, and GO (Gene Ontology)^92^ via Blast2GO program^93^. The reads were mapped on the non-redundant reference using Bowtie2^94^. Genes with count per million (CPM) values less than 1 in all groups were excluded from downstream analysis^95^. Normalization and differential expression were carried out using DESeq2^96^ in R environment^97^. A pairwise comparison was performed. Differentially expressed genes were those with their absolute value of log_2_ fold change ≥ 1, with *p value* < 0.05. Non-differentially expressed genes were clustered as one group whereas significant differentially expressed genes were clustered based on their altered expressed transcripts using unsupervised hierarchical clustering and quality threshold clustering (QTC) method. QTC was conducted to determine gene expression patterns using MeV with following criteria (1) diameter of 0.5 and (2) a minimum of 50 cluster members^98^. To understand biological functions of each gene cluster, gene set enrichment analysis was performed using reactome pathway analysis^99^.

### Validation of gene expression by quantitative real-time PCR (qPCR) analysis

qPCR was performed to validate gene expression patterns obtained from RNA-seq. Complementary DNA was synthesized from each RNA sample using ImPromII Reverse Transcription System kit (Promega, USA) according to the manufacturer’s protocol. Purity and concentration of cDNA samples were determined by using NanoDrop (ND-8000) spectrophotometer. Eleven immune-related genes were selected for qPCR validation. Specific primer of each gene was designed using Primer Premier Program (Table S2). Each qPCR reaction contained 100 ng of cDNA template, 0.2 µM of each primer and 1X SYBR Green SsoAdvanced (BioRad). The cycle parameters were as follows; initial denaturation at 95 °C for 30 s, followed by 40 cycles of 95 °C for 15 s, 56 °C for 30 s, 72 °C for 30 s and extension at 72 °C for 1 min. The specificity of each PCR product was confirmed by melting curve analysis when temperature was reducing from 65 to 95 °C at 0.5 °C increment with a continuous fluorescent reading. The expression profile of each gene was calculated using 2^−**ΔΔCT**^ method^100^. Relative gene expression analysis was normalized to that the housekeeping gene (*Elongation factor 1α*, *EF1α*) as an internal control. All qPCRs were performed in three biological replicates (*n* = *3*). The relative expression level of each gene from different shrimp growth stages were statistically tested using one-way analysis of variance (ANOVA) followed by Duncan’s new multiple range test in IBM SPSS statistics 23.0.

## Supplementary Information

Supplementary Figure and Table.

Supplementary Table.

## Data Availability

The transcriptome dataset was deposited to BioProject at NCBI under accession Number PRJNA688806.
