# Supplementary material for: Transcriptomic analysis of the black tiger shrimp (Penaeus monodon) reveals insights into immune development in their early life stages
Source: Sci Rep. 2021 Jul 6;11:13881. doi: 10.1038/s41598-021-93364-9 (PMC8260638; doi:10.1038/s41598-021-93364-9)
Supplement: Supplementary file 1 — Supplementary Figure and Table. [file 41598_2021_93364_MOESM1_ESM.pdf]

Transcriptomic analysis of the black tiger shrimp (*Penaeus monodon*) reveals insights into immune development in their early life stages,

Pacharaporn Angthong

Tanaporn Uengwetwanit

Sopacha Arayamethakorn

Wanilada Rungrassamee

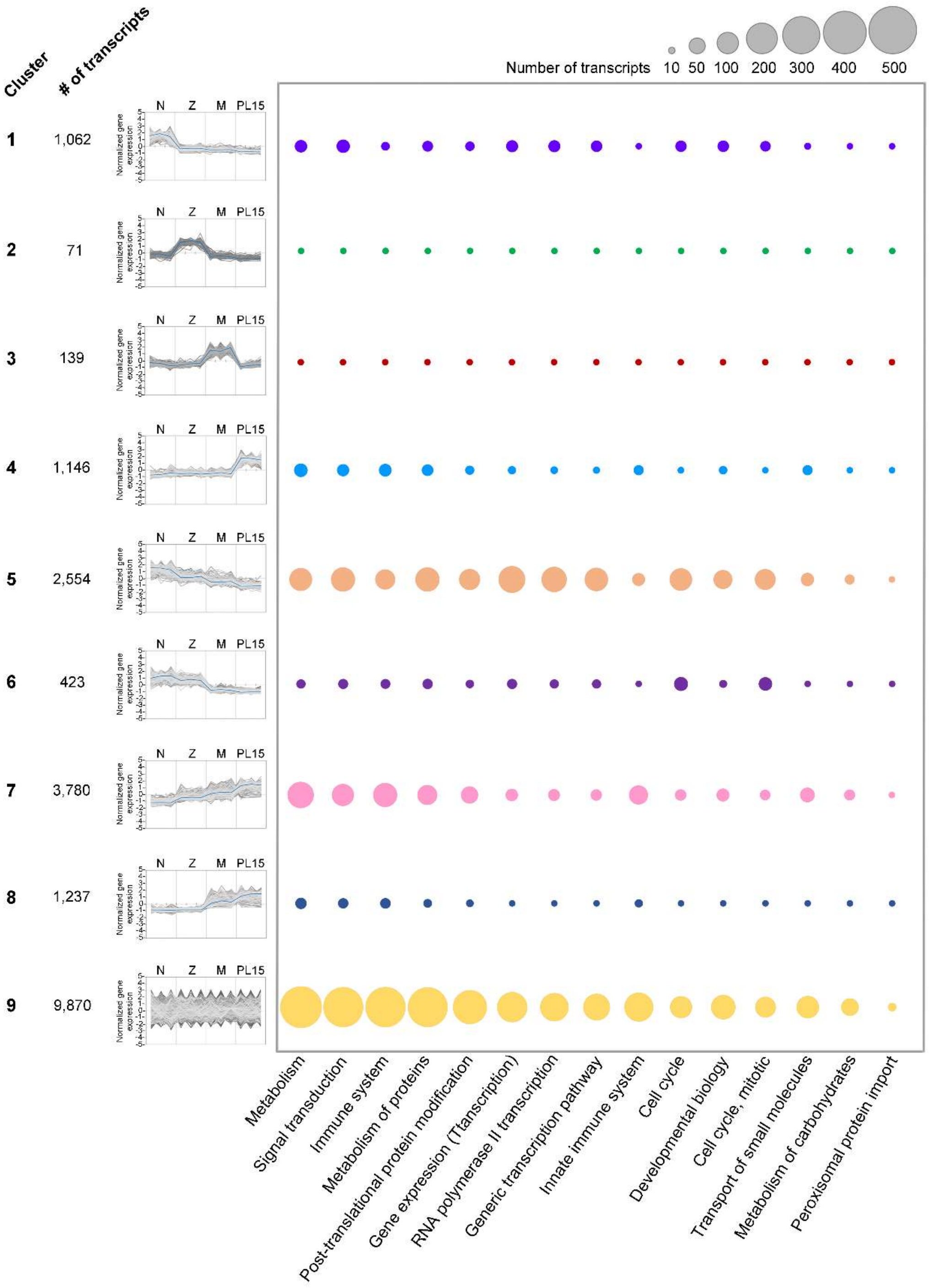

Figure S1. Gene clustering and reactome pathway enrichment analysis across shrimp growth stages (nauplius, zoea, mysis and 15-day-old postlarva). Cluster 1-4 shows the pattern of highly expressed transcripts in nauplius, zoea, mysis and 15-day-old postlarva, respectively. Cluster 5-6 shows decreasing pattern of gene expression, and cluster 7-8 shows increasing pattern of gene expression. Cluster 9 shows the pattern of unchanged in gene expression across all life stages.

Table S2. Primers used in quantitative real-time PCR analysis.

**Gene**	**ID**	**Primer sequence (5' to 3')**	**Product size (bp)**
Crustin Pm4	Crustin Pm4	F: TAAGTGGTGGTTTGGGTGGT<br>R: GGTTTGGTGCCAACAGGTAT	181
Antiviral protein	Antiviral protein	F: TACACGATTGTTGCCCATGT<br>R: ACATGCCTCTCTGGCTTCAT	120
Penlectin5-2	PL5-2	F: ACGTGATCTTCCCGTACGAC<br>R: AAGTCCTCCTGCTCAGCGTA	124
C-type lectin4	C-type lectin4	F: TACTTCAACGACGCCAACTG<br>R: AAGACCAAGCTCGTCAGAGG	160
Serpin3	Serpin3	F: TTCTGGCAACCGAGATCTTT<br>R: ACATGAATGGATGGTCAGCA	161
Prophenoloxidase activating factor1	PPAF1	F: CCTCCATCCTTCCTTCAACA<br>R: AGCCAATCACGATAGCGTCT	167
Prophenoloxidase activating factor2	PPAF2	F: GTTGCCCAGATCAGAAGGAA<br>R: ATCGTCATCCAAGGCAATTC	148
Hemolymph clottable protein	Hemolymph clottable protein	F: TTCAGATCCACAGCAGCAAG<br>R: AGGTCTGGTTGGTGAAGGTG	177
Heat shock protein70	HSP70	F: TACTCCTGCGTCGGAGTCTT<br>R: ATCGTTTGGCATCAAACACA	175
Superoxide dismutase (Mn)	MnSOD	F: TTGCCCATATCAATGCTGAA<br>R: TGATGCTTTGTGTGGTGGAT	130
Toll-like receptor1	Toll-like receptor1	F: GATAGTGCCCAGGCGTATGT<br>R: AATACCTCGCCAAACGACAC	170
